# Supplementary material for: Patterns of compliance with COVID-19 preventive behaviours: a latent class analysis of 20 000 UK adults
Source: J Epidemiol Community Health. 2021 Sep 14;76(3):247–53. doi: 10.1136/jech-2021-216876 (PMC8449842; doi:10.1136/jech-2021-216876)
Supplement: Supplementary data [file jech-2021-216876supp001.pdf]

SUPPLEMENTARY INFORMATION

POLICY ENVIRONMENT

The period 17 November to 23 December 2020 overlaps with the second wave of COVID-19 in the UK in which there were several changes to COVID-19 related rules. The rules differed somewhat between the nations of England, Scotland, Wales, and Northern Ireland, though mask wearing in indoor public spaces was legally mandated in each nation. On 17 November, England was two weeks into a four-week national lockdown in which non-essential business were closed, employees were asked to work from home where possible, and household mixing was banned. A new three-tier system was put in place from 2 December when lockdown ended. Several areas were placed in the highest tier. In Wales, rules were more relaxed, following a three week "circuit breaker" lockdown that ended on 9 November. Two households were allowed to form bubbles, non-essential shops were open and groups of four people were allowed to meet in indoor public spaces. In Scotland, a five-tier localised system was in place. On 20 November, eleven council areas were placed in tier 4 and non-essential travel between Scotland and England was banned. Northern Ireland was in a circuit breaker lockdown, due to expire on 27 November. Following this, strict rules are put in place, with non-essential shop barred from opening and limited household mixing allowed.

On 24 November, the leads of the four national Governments announced plans to allow mixing of three households for five days between 23-27 December (22-28 December in Northern Ireland), though these plans were later reduced and restricted in some areas. On 2 December, the UK approved the Pfizer/BioNTech COVID-19 vaccine with the first vaccination provided on 8 December. 137,987 people were vaccinated in the first week. On 14 December, a new, more virulent variant of SARS-CoV-2 is identified. Areas in the South East of England are placed into lockdown from 19 December, with Christmas bubbles cancelled in these areas and relaxation elsewhere in England limited to Christmas day. Supplementary Figure S1 shows 7-day COVID-19 caseloads and confirmed deaths, along with the Oxford Policy Tracker [1], a numerical summary of policy stringency, across the study period.

MEASURES

Compliance Behaviour

Compliance behaviour was measured with the following six question:

In the last 7 days, to what extent have you been following the behaviours below?
1. Washing your hands thoroughly with soap and water or using hand sanitising gel after any possible contact with other people outside of your household or shared surfaces<br>2. Wearing a face mask or other face covering where it is currently recommended<br>3. Maintaining the recommended distance from people not in your household/bubble<br>4. Meeting up with MORE THAN the recommended number of people from other households OUTDOORS<br>5. Meeting up with MORE THAN the recommended number of people from other households INDOORS<br>6. Following other rules relevant to the tier or level of lockdown currently active in your area

The response categories were: never, rarely, occasionally, frequently, always, not applicable. We treated "not applicable" as a missing value. Items on household mixing were phrased such that higher scores indicated lower compliance. We reverse coded these for consistency with the other behaviours.

Predictors of Compliance Behaviour

Demographics and Socio-Economic Position

We included the demographic variables for country of residence (England/Wales/NI vs Scotland) and tier (Lockdown, Tier 1, Tier 2, Tier 3, Wales, Northern Ireland), gender (male, female), ethnicity (White or Non-White) and age (18-29, 30-44, 45-59, 60+). We also included variables for socio-economic position (SEP): annual income (< £16k, £16k - £30k, £30k - £60k, £60k - £90k, £90k +), keyworker status (yes, no), education level (GCSE or below, A-levels or equivalent, degree or above), household overcrowding (< 1 person per room, 1+ persons per room), and living arrangement (alone, with adult but no child, with child). Besides tier, which was measure at same data collection as compliance behaviour, each variable was measured at baseline interview. Keyworker status was was defined as employment in any of the following roles: health, social care or relevant related support worker; teacher or childcare worker still travelling in to work; transport worker still travelling in to work; food chain worker (e.g. production, sale, delivery); key public services worker (e.g. justice staff, religious staff, public service journalist or mortuary worker); local or national government worker delivering essential public services; utility worker (e.g. energy, sewerage, postal service); public safety or national security worker; worker involved in medicines or protective equipment production or distribution. Note, the coding of the country of residence and tier variables to ensure non-collinearity.

We also assessed neighbourhood crowding with items collected between 09-16 July. Neighbourhood crowding was measured using three items on satisfaction with neighbourhood traffic density, noise, and levels of crowding. Each was measured on a three-point scale (categories as above), which we reverse coded and summed into a single score (range 3 – 9). Higher values indicate less crowding.

Personality Traits

Personality was measured at baseline interview using the Big Five Inventory [BFI-2; 2], which measures five domains and 15 facets: openness (intellectual curiosity, aesthetic sensitivity, and creative imagination), conscientiousness (organisation, productiveness, and responsibility), extraversion (sociability, assertiveness, and energy level), agreeableness (compassion, respectfulness, and trust) and neuroticism (anxiety, depression, and emotional volatility). Each item was scored on a 5-point scale (1 = "strongly disagree", 5 = "strongly agree"). We use the sum Likert score for each domain (range 3 - 15). High values indicate high levels of the trait.

Locus of control was measured between 04 – 11 June using the 6-item Locus of Control Scale developed by the University of Washington Beyond High School Project [3], and captures generalized expectancies about whether individuals can (internal) or cannot (external) control events and outcomes in their lives. Responses were rated on a four-point scale ranging from (1 = "strongly agree", 4 = "strongly disagree"). We used the sum score of responses, with items coded such that higher values indicate more external locus of control.

Risk-taking was measured between 23-30 July with one item from the Dohmen Risk Taking Scale [4]. Respondents rated the extent to which they generally see themselves as a person who is fully prepared to take risks, rated on an 11-point scale (0 = “not at all willing to take risks” to 10 = “very willing to take risks”).

Empathy was assessed between 11-18 July using subscales for empathic concern from the Interpersonal Reactivity Index [IRI; 5]. (Subscales for fantasy and personal distress were not administered.) Empathic concern (also known as emotional empathy) consists of 7 items and captures feelings of warmth, concern, and compassion for others. Items were rated on a five-point scale ranging (1 = “does not describe me well”, 5 = “describes me very well”). We used the sum Likert score, coding items such that higher scores indicate higher empathy (range 0 - 28).

Health and Confidence in Government

We included variables for long-term physical health conditions (0, 1, 2+) using a multiple-choice question on medical conditions. Included conditions were high blood pressure, diabetes, heart disease, lung disease, cancer, any other clinically-diagnosed chronic physical health conditions, or any disability. Psyhciatric diagnosis (yes, no) was with the same multiple choice question using items on clinically diagnosed depression, clinically diagnosed anxiety, and any other clinically diagnosed mental health problem. The data was collected at baseline interview.

Mental health during first lockdown vis-à-vis prior to the pandemic was measured with a single item question (“How do you feel your mental health was affected during lockdown in April/May?”). The response categories were: my mental health got worse compared to before Covid-19; My mental health was about the same; my mental health got better compared to before Covid-19. This variable was included in the survey between 18-25 June. Finally, confidence in government was measured with a single item question (“How much confidence do you have in the CENTRAL UK GOVERNMENT that they can handle Covid-19 well?”). Responses were scored on a Likert scale (1. None at all; 7. Lots). This item has been asked at each follow-up. We used responses from the same data collection as compliance behaviours were measured.

FIGURES

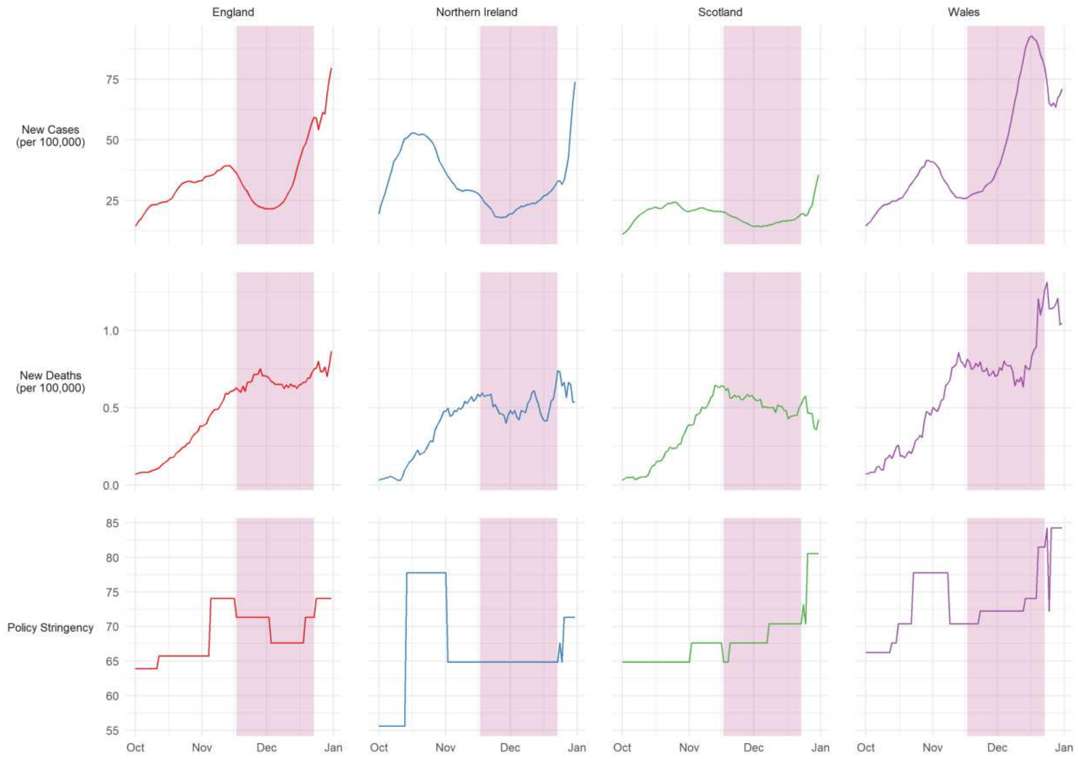

Figure S1: 7-day COVID-19 confirmed cases, COVID-19 deaths, and policy stringency by country. Source: Hale et al. [1]. Pink shaded band represents period in which compliance behaviours were measured.

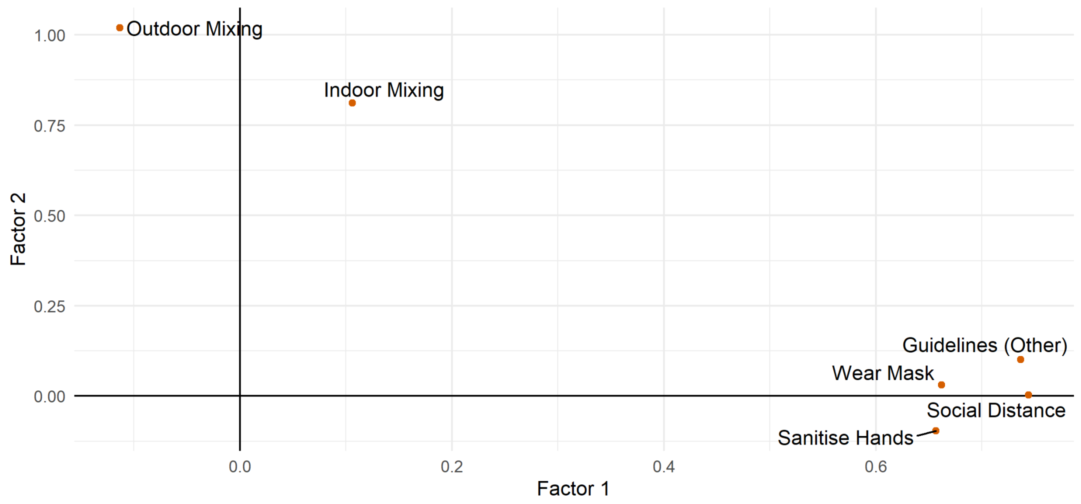

Figure S2: Factor loadings derived from exploratory factor analysis of six compliance items using polychoric correlations and promax rotation.

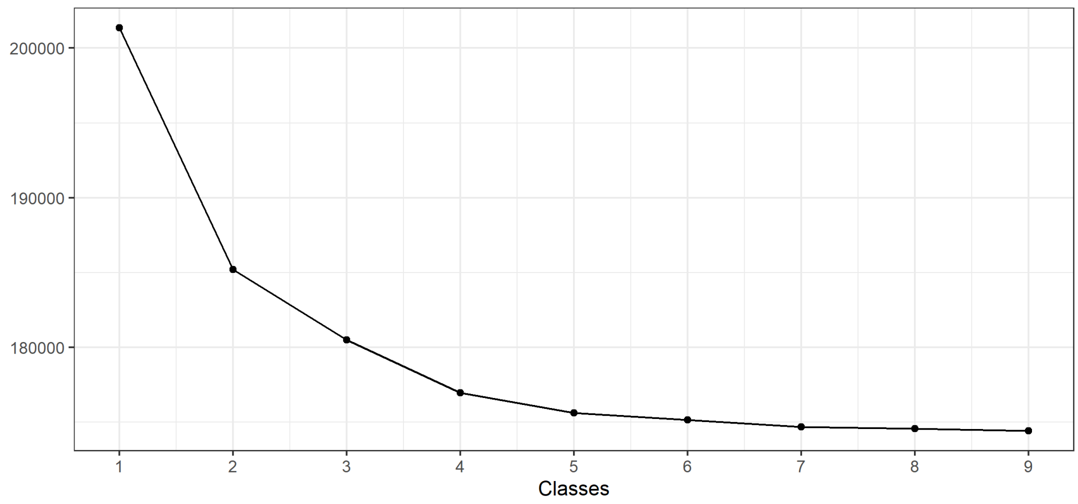

Figure S3: BIC elbow plot for 1-9 latent class solutions.

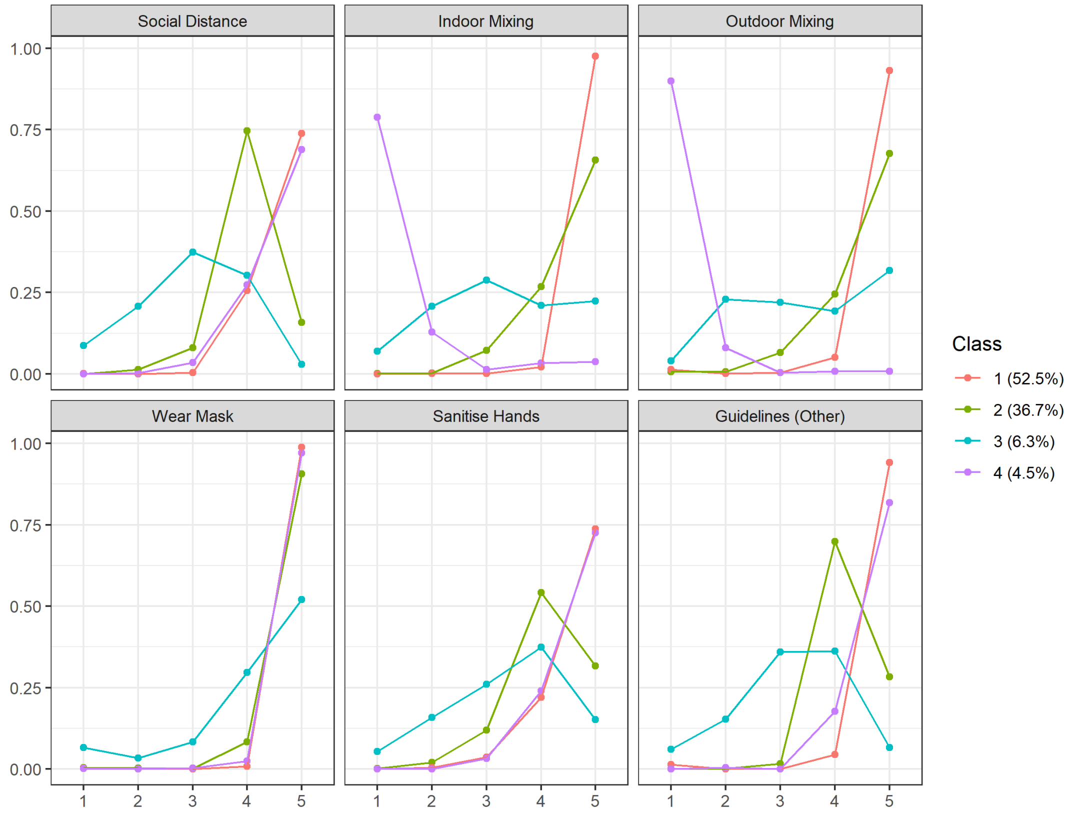

Figure S4: Predicted probability of compliance level by compliance behaviour and latent class

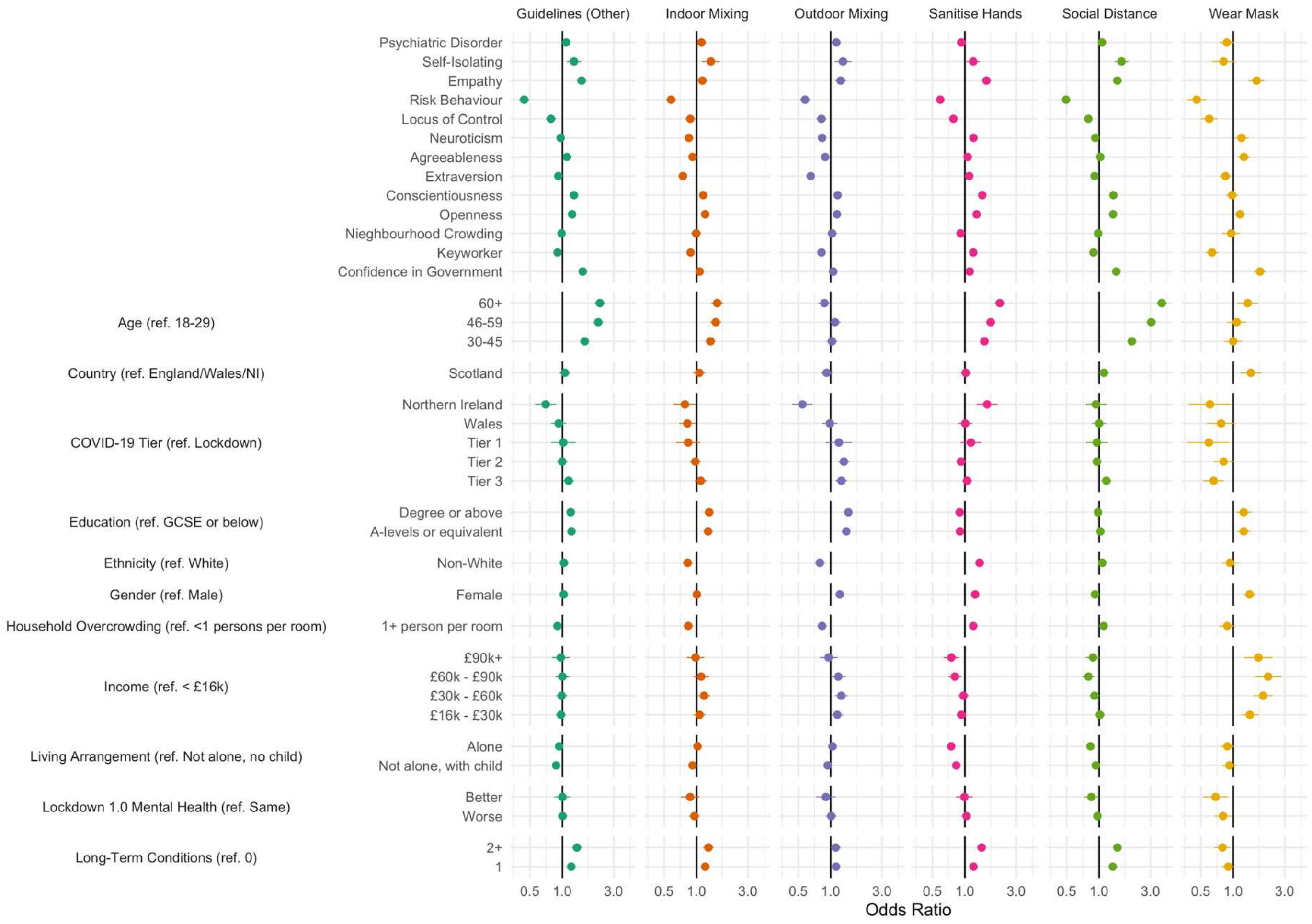

Figure S5: Results of multivariate ordinal regressions predicting compliance behaviour according to participant characteristics. Regressions use multiply imputed data (40 datasets).

TABLES

Table S1: Sample descriptive statistics.

	Variable	Unweighted Observed	Missing %	Weighted Imputed
	n	20,947		20,947
Social Distance	Never	61 (0.29%)	0%	123.62 (0.59%)
	Rarely	195 (0.93%)		383.22 (1.83%)
	Occasionally	793 (3.79%)		1,189.95 (5.68%)
	Frequently	9,124 (43.56%)		9,217.73 (44%)
	Always	10,774 (51.43%)		10,032.48 (47.89%)
Indoor Mixing	Always	766 (3.66%)	0%	835.77 (3.99%)
	Frequently	280 (1.34%)		415.04 (1.98%)
	Occasionally	798 (3.81%)		956.44 (4.57%)
	Rarely	2,334 (11.14%)		2,616.46 (12.49%)
	Never	16,769 (80.05%)		16,123.29 (76.97%)
Outdoor Mixing	Always	990 (4.73%)	0%	1,078.18 (5.15%)
	Frequently	278 (1.33%)		443.85 (2.12%)
	Occasionally	760 (3.63%)		837.68 (4%)
	Rarely	2,411 (11.51%)		2,706.48 (12.92%)
	Never	16,508 (78.81%)		15,880.80 (75.81%)
Wear Mask	Never	99 (0.47%)	0%	158.83 (0.76%)
	Rarely	46 (0.22%)		73.00 (0.35%)
	Occasionally	83 (0.4%)		136.23 (0.65%)
	Frequently	913 (4.36%)		1,148.91 (5.48%)
	Always	19,806 (94.55%)		19,430.03 (92.76%)
Sanitise Hands	Never	81 (0.39%)	0%	103.65 (0.49%)
	Rarely	285 (1.36%)		411.35 (1.96%)
	Occasionally	1,341 (6.4%)		1,688.16 (8.06%)
	Frequently	7,138 (34.08%)		7,310.35 (34.9%)

	Variable	Unweighted Observed	Missing %	Weighted Imputed
	Always	12,102 (57.77%)		11,433.49 (54.58%)
Other Guidelines	Never	187 (0.89%)	0%	233.44 (1.11%)
	Rarely	132 (0.63%)		238.43 (1.14%)
	Occasionally	392 (1.87%)		597.38 (2.85%)
	Frequently	5,861 (27.98%)		6,487.19 (30.97%)
	Always	14,375 (68.63%)		13,390.57 (63.93%)
	Confidence in Government	3.19 (1.83)		3.29 (1.89)
COVID-19 Tier	Lockdown	3,256 (15.67%)	0.81%	3,424.35 (16.35%)
	Tier 3	6,019 (28.97%)		6,896.29 (32.92%)
	Tier 2	7,985 (38.43%)		8,625.04 (41.18%)
	Tier 1	425 (2.05%)		418.82 (2%)
	Wales	2,892 (13.92%)		1,085.69 (5.18%)
	Northern Ireland	201 (0.97%)		496.81 (2.37%)
Country	England/Wales/NI	19,679 (93.95%)	0%	19,247.51 (91.89%)
	Scotland	1,268 (6.05%)		1,699.49 (8.11%)
Gender	Male	5,374 (25.66%)	0%	9,849.22 (47.02%)
	Female	15,573 (74.34%)		11,097.78 (52.98%)
Ethnicity	White	20,264 (96.74%)	0%	18,834.33 (89.91%)
	Non-White	683 (3.26%)		2,112.67 (10.09%)
Age	18-29	804 (3.84%)	0%	3,169.20 (15.13%)
	30-45	3,970 (18.95%)		5,527.74 (26.39%)
	46-59	6,680 (31.89%)		5,406.59 (25.81%)
	60+	9,493 (45.32%)		6,843.47 (32.67%)
Keyworker	No	16,846 (80.42%)	0%	16,482.43 (78.69%)
	Yes	4,101 (19.58%)		4,464.57 (21.31%)
Education	GCSE or below	2,993 (14.29%)	0%	6,718.58 (32.07%)
	A-levels or equivalent	3,531 (16.86%)		6,758.60 (32.27%)

	Variable	Unweighted Observed	Missing %	Weighted Imputed
	Degree or above	14,423 (68.85%)		7,469.81 (35.66%)
Income	< £16k	2,696 (14.34%)	10.24%	3,703.64 (17.68%)
	£16k - £30k	4,972 (26.45%)		6,016.19 (28.72%)
	£30k - £60k	6,633 (35.28%)		7,140.88 (34.09%)
	£60k - £90k	2,689 (14.3%)		2,554.19 (12.19%)
	£90k+	1,811 (9.63%)		1,532.10 (7.31%)
Living Arrangement	Not alone, no child	12,314 (58.79%)	0%	12,475.06 (59.56%)
	Not alone, with child	4,120 (19.67%)		4,528.07 (21.62%)
	Alone	4,513 (21.54%)		3,943.87 (18.83%)
Household Overcrowding	<1 persons per room	19,597 (93.56%)	0%	18,319.81 (87.46%)
	1+ person per room	1,350 (6.44%)		2,627.19 (12.54%)
	Nieghbourhood Crowding	7.03 (1.84)	24.48%	6.95 (1.85)
Lockdown 1.0 Mental Health	Same	10,249 (62.2%)	21.33%	12,448.18 (59.43%)
	Worse	5,001 (30.35%)		6,858.43 (32.74%)
	Better	1,228 (7.45%)		1,640.39 (7.83%)
Personality Traits	Openness	15.3 (3.23)	0%	14.75 (3.26)
	Conscientiousness	16.08 (2.88)	0%	15.78 (2.9)
	Extraversion	12.88 (4.25)	0%	12.51 (4.28)
	Agreeableness	15.59 (3.01)	0%	15.44 (3.13)
	Neuroticism	10.9 (4.21)	0%	11.27 (4.37)
	External Locus of Control	12.14 (2.58)	20.82%	12.53 (2.67)
	Risk Behaviour	4.38 (2.33)	25.4%	4.42 (2.36)
	Empathy	20.67 (4.6)	22.33%	20 (4.79)
Self-Isolating	No	19,010 (93.4%)	2.84%	19,640.32 (93.76%)
	Yes	1,343 (6.6%)		1,306.68 (6.24%)
Psychiatric Disorder	No	17,997 (85.92%)	0%	17,407.99 (83.1%)
	Yes	2,950 (14.08%)		3,539.01 (16.9%)

	Variable	Unweighted Observed	Missing %	Weighted Imputed
Long-Term Conditions	0	11,935 (56.98%)	0%	12,247.76 (58.47%)
	1	5,852 (27.94%)		5,579.65 (26.64%)
	2+	3,160 (15.09%)		3,119.59 (14.89%)

Table S2: Sample descriptive statistics by most likely latent class.

	Variable	Class 1	Class 2	Class 3	Class 4
	n	11,780.40	7,063.10	1,187.06	916.44
Social Distance	Never	9.45 (0.08%)	-	114.18 (9.62%)	-
	Rarely	-	110.50 (1.56%)	270.31 (22.77%)	2.41 (0.26%)
	Occasionally	47.94 (0.41%)	647.46 (9.17%)	462.82 (38.99%)	31.73 (3.46%)
	Frequently	3,450.72 (29.29%)	5,197.75 (73.59%)	311.22 (26.22%)	258.04 (28.16%)
	Always	8,272.30 (70.22%)	1,107.39 (15.68%)	28.53 (2.4%)	624.26 (68.12%)
Indoor Mixing	Always	-	6.78 (0.1%)	89.51 (7.54%)	739.48 (80.69%)
	Frequently	9.56 (0.08%)	7.54 (0.11%)	272.27 (22.94%)	125.67 (13.71%)
	Occasionally	9.46 (0.08%)	590.38 (8.36%)	345.10 (29.07%)	11.49 (1.25%)
	Rarely	148.98 (1.26%)	2,194.64 (31.07%)	235.42 (19.83%)	37.43 (4.08%)
	Never	11,612.40 (98.57%)	4,263.76 (60.37%)	244.76 (20.62%)	2.36 (0.26%)
Outdoor Mixing	Always	168.02 (1.43%)	33.51 (0.47%)	45.23 (3.81%)	831.42 (90.72%)
	Frequently	11.54 (0.1%)	62.69 (0.89%)	298.22 (25.12%)	71.39 (7.79%)
	Occasionally	29.78 (0.25%)	546.53 (7.74%)	258.43 (21.77%)	2.94 (0.32%)
	Rarely	539.32 (4.58%)	1,930.61 (27.33%)	230.64 (19.43%)	5.92 (0.65%)
	Never	11,031.73 (93.64%)	4,489.77 (63.57%)	354.54 (29.87%)	4.76 (0.52%)

	Variable	Class 1	Class 2	Class 3	Class 4
Wear Mask	Never	30.14 (0.26%)	39.91 (0.57%)	87.16 (7.34%)	1.62 (0.18%)
	Rarely	4.31 (0.04%)	23.49 (0.33%)	45.20 (3.81%)	-
	Occasionally	9.53 (0.08%)	13.87 (0.2%)	109.67 (9.24%)	3.16 (0.34%)
	Frequently	84.96 (0.72%)	665.94 (9.43%)	374.12 (31.52%)	23.89 (2.61%)
	Always	11,651.46 (98.91%)	6,319.89 (89.48%)	570.91 (48.09%)	887.76 (96.87%)
Sanitise Hands	Never	17.30 (0.15%)	13.70 (0.19%)	72.65 (6.12%)	-
	Rarely	23.09 (0.2%)	186.21 (2.64%)	201.20 (16.95%)	0.86 (0.09%)
	Occasionally	507.95 (4.31%)	839.69 (11.89%)	305.29 (25.72%)	35.22 (3.84%)
	Frequently	2,882.83 (24.47%)	3,761.64 (53.26%)	439.40 (37.02%)	226.47 (24.71%)
	Always	8,349.23 (70.87%)	2,261.87 (32.02%)	168.51 (14.2%)	653.88 (71.35%)
Other Guidelines	Never	153.87 (1.31%)	-	79.57 (6.7%)	-
	Rarely	18.43 (0.16%)	10.87 (0.15%)	209.13 (17.62%)	-
	Occasionally	-	110.53 (1.56%)	486.65 (41%)	0.20 (0.02%)
	Frequently	314.56 (2.67%)	5,654.68 (80.06%)	352.93 (29.73%)	165.01 (18.01%)
	Always	11,293.54 (95.87%)	1,287.03 (18.22%)	58.77 (4.95%)	751.23 (81.97%)
	Confidence in Government	3.42 (1.95)	3.15 (1.76)	2.65 (1.75)	3.56 (1.96)
COVID-19 Tier	Lockdown	1,866.94 (15.85%)	1,219.93 (17.27%)	208.98 (17.6%)	128.51 (14.02%)

	Variable	Class 1	Class 2	Class 3	Class 4
	Tier 3	4,033.94 (34.24%)	2,193.97 (31.06%)	349.65 (29.46%)	318.72 (34.78%)
	Tier 2	4,739.27 (40.23%)	2,994.06 (42.39%)	523.12 (44.07%)	368.60 (40.22%)
	Tier 1	250.89 (2.13%)	124.77 (1.77%)	22.91 (1.93%)	20.25 (2.21%)
	Wales	639.99 (5.43%)	347.95 (4.93%)	48.93 (4.12%)	48.81 (5.33%)
	Northern Ireland	249.37 (2.12%)	182.43 (2.58%)	33.47 (2.82%)	31.54 (3.44%)
Country	England/Wales/NI	10,849.03 (92.09%)	6,440.25 (91.18%)	1,115.12 (93.94%)	843.11 (92%)
	Scotland	931.37 (7.91%)	622.85 (8.82%)	71.94 (6.06%)	73.32 (8%)
Gender	Male	5,344.62 (45.37%)	3,349.11 (47.42%)	626.77 (52.8%)	528.72 (57.69%)
	Female	6,435.78 (54.63%)	3,713.99 (52.58%)	560.29 (47.2%)	387.72 (42.31%)
Ethnicity	White	10,654.43 (90.44%)	6,349.89 (89.9%)	1,034.94 (87.19%)	795.07 (86.76%)
	Non-White	1,125.97 (9.56%)	713.22 (10.1%)	152.12 (12.81%)	121.36 (13.24%)
Age	18-29	1,334.02 (11.32%)	1,440.94 (20.4%)	341.84 (28.8%)	52.40 (5.72%)
	30-45	2,862.69 (24.3%)	2,078.89 (29.43%)	363.15 (30.59%)	223.01 (24.33%)
	46-59	3,284.26 (27.88%)	1,647.23 (23.32%)	244.55 (20.6%)	230.54 (25.16%)
	60+	4,299.43 (36.5%)	1,896.04 (26.84%)	237.52 (20.01%)	410.48 (44.79%)
Keyworker	No	9,461.27 (80.31%)	5,432.78 (76.92%)	871.02 (73.38%)	717.36 (78.28%)

	Variable	Class 1	Class 2	Class 3	Class 4
	Yes	2,319.13 (19.69%)	1,630.32 (23.08%)	316.04 (26.62%)	199.08 (21.72%)
Education	GCSE or below	3,970.63 (33.71%)	1,984.39 (28.1%)	400.81 (33.76%)	362.76 (39.58%)
	A-levels or equivalent	3,825.68 (32.47%)	2,298.73 (32.55%)	348.59 (29.37%)	285.61 (31.16%)
	Degree or above	3,984.09 (33.82%)	2,779.99 (39.36%)	437.66 (36.87%)	268.07 (29.25%)
Income	< £16k	2,092.37 (17.76%)	1,153.56 (16.33%)	264.06 (22.25%)	193.64 (21.13%)
	£16k - £30k	3,529.09 (29.96%)	1,922.61 (27.22%)	308.45 (25.98%)	256.04 (27.94%)
	£30k - £60k	4,019.96 (34.12%)	2,477.60 (35.08%)	373.06 (31.43%)	270.25 (29.49%)
	£60k - £90k	1,361.05 (11.55%)	952.11 (13.48%)	139.49 (11.75%)	101.54 (11.08%)
	£90k+	777.93 (6.6%)	557.22 (7.89%)	101.98 (8.59%)	94.96 (10.36%)
Living Arrangement	Not alone, no child	7,192.11 (61.05%)	4,062.25 (57.51%)	644.92 (54.33%)	575.78 (62.83%)
	Not alone, with child	2,385.20 (20.25%)	1,687.05 (23.89%)	287.08 (24.18%)	168.74 (18.41%)
	Alone	2,203.09 (18.7%)	1,313.81 (18.6%)	255.06 (21.49%)	171.92 (18.76%)
Household Overcrowding	<1 persons per room	10,512.03 (89.23%)	6,047.53 (85.62%)	970.34 (81.74%)	789.91 (86.19%)
	1+ person per room	1,268.37 (10.77%)	1,015.57 (14.38%)	216.72 (18.26%)	126.53 (13.81%)
	Nieghbourhood Crowding	6.95 (1.87)	6.95 (1.82)	6.91 (1.8)	7.03 (1.87)
Lockdown 1.0 Mental Health	Same	7,168.37 (60.85%)	4,153.77 (58.81%)	510.84 (43.03%)	615.20 (67.13%)

	Variable	Class 1	Class 2	Class 3	Class 4
	Worse	3,748.86 (31.82%)	2,340.89 (33.14%)	518.38 (43.67%)	250.30 (27.31%)
	Better	863.17 (7.33%)	568.44 (8.05%)	157.84 (13.3%)	50.94 (5.56%)
	Openness	14.86 (3.31)	14.65 (3.1)	14.2 (3.42)	14.79 (3.5)
	Conscientiousness	16.04 (2.9)	15.39 (2.79)	15.29 (3.07)	15.99 (3.05)
	Extraversion	12.46 (4.28)	12.57 (4.22)	12.38 (4.6)	12.84 (4.29)
	Agreeableness	15.63 (3.13)	15.18 (3.01)	14.87 (3.35)	15.77 (3.41)
	Neuroticism	11.27 (4.4)	11.24 (4.31)	11.54 (4.45)	11.11 (4.37)
	External Locus of Control	12.43 (2.68)	12.59 (2.59)	13.24 (2.99)	12.53 (2.64)
	Risk Behaviour	4.04 (2.35)	4.81 (2.21)	5.74 (2.48)	4.45 (2.41)
	Empathy	20.49 (4.76)	19.43 (4.7)	18.54 (4.99)	20.02 (4.8)
Self-Isolating	No	10,863.39 (92.22%)	6,788.61 (96.11%)	1,149.35 (96.82%)	838.96 (91.55%)
	Yes	917.01 (7.78%)	274.49 (3.89%)	37.71 (3.18%)	77.47 (8.45%)
Psychiatric Disorder	No	9,760.18 (82.85%)	5,959.08 (84.37%)	912.82 (76.9%)	775.92 (84.67%)
	Yes	2,020.22 (17.15%)	1,104.03 (15.63%)	274.24 (23.1%)	140.52 (15.33%)
Long-Term Conditions	0	6,324.24 (53.68%)	4,667.52 (66.08%)	780.92 (65.79%)	475.09 (51.84%)
	1	3,413.22 (28.97%)	1,648.78 (23.34%)	249.66 (21.03%)	267.99 (29.24%)
	2+	2,042.94 (17.34%)	746.81 (10.57%)	156.48 (13.18%)	173.36 (18.92%)

Table S3: Results of multinomial regression model predicting latent class by individual characteristics. Models use 3-step procedure and multiply imputed data (40 datasets). Reference class is largest class, Class 1.

	Variable	Class 2	Class 3	Class 4
	Confidence in Government	0.86 (0.8, 0.93)	0.6 (0.49, 0.73)	1.02 (0.89, 1.17)
COVID-19 Tier (ref. Lockdown)	Tier 3	0.87 (0.67, 1.13)	0.64 (0.35, 1.15)	1.02 (0.62, 1.67)
	Tier 2	1.06 (0.82, 1.38)	0.82 (0.47, 1.43)	0.97 (0.61, 1.55)
	Tier 1	0.94 (0.56, 1.6)	1.12 (0.35, 3.58)	0.96 (0.4, 2.32)
	Wales	1.09 (0.81, 1.47)	0.74 (0.35, 1.56)	0.93 (0.55, 1.56)
	Northern Ireland	1.54 (0.78, 3.03)	1.47 (0.41, 5.28)	1.9 (0.75, 4.79)
Country (ref. England/Wales/NI)	Scotland	1.13 (0.84, 1.53)	0.57 (0.27, 1.19)	1.08 (0.68, 1.71)
Gender (ref. Male)	Female	1 (0.84, 1.17)	0.72 (0.49, 1.04)	0.63 (0.47, 0.84)
Ethnicity (ref. White)	Non-White	0.84 (0.61, 1.16)	1.02 (0.57, 1.83)	1.81 (1.12, 2.92)
Age (ref. 18-29)	30-45	0.57 (0.42, 0.78)	0.34 (0.2, 0.58)	2.23 (0.81, 6.17)
	46-59	0.4 (0.3, 0.55)	0.2 (0.11, 0.36)	1.96 (0.71, 5.35)
	60+	0.36 (0.26, 0.5)	0.16 (0.09, 0.31)	2.76 (1.01, 7.55)
	Keyworker	1.11 (0.93, 1.34)	1.27 (0.86, 1.89)	1.28 (0.91, 1.8)
Education (ref. GCSE or below)	A-levels or equivalent	0.95 (0.78, 1.16)	0.5 (0.32, 0.78)	0.89 (0.63, 1.26)
	Degree or above	1 (0.82, 1.22)	0.51 (0.32, 0.79)	0.8 (0.56, 1.13)

	Variable	Class 2	Class 3	Class 4
Income (ref. < £16k)	£16k - £30k	0.98 (0.76, 1.25)	0.72 (0.43, 1.19)	0.77 (0.51, 1.17)
	£30k - £60k	0.98 (0.75, 1.27)	0.7 (0.4, 1.22)	0.79 (0.51, 1.24)
	£60k - £90k	0.94 (0.69, 1.29)	0.63 (0.3, 1.32)	0.99 (0.56, 1.75)
	£90k+	1 (0.69, 1.44)	0.92 (0.45, 1.89)	1.67 (0.92, 3.03)
Living Arrangement (ref. Not alone, no child)	Not alone, with child	1.18 (0.97, 1.44)	1.32 (0.85, 2.03)	0.84 (0.55, 1.27)
	Alone	1.14 (0.94, 1.38)	1.37 (0.92, 2.03)	0.97 (0.7, 1.34)
Household Overcrowding (ref. <1 persons per room)	1+ person per room	1.11 (0.85, 1.46)	1.29 (0.79, 2.09)	1.54 (0.95, 2.48)
	Nieghbourhood Crowding	1.02 (0.93, 1.11)	1.12 (0.93, 1.35)	1.05 (0.89, 1.24)
Lockdown 1.0 Mental Health (ref. Same)	Worse	0.85 (0.7, 1.04)	1.58 (1.05, 2.38)	0.84 (0.56, 1.27)
	Better	0.82 (0.59, 1.15)	2.24 (1.14, 4.38)	0.81 (0.38, 1.74)
	Openness	0.9 (0.83, 0.97)	0.7 (0.59, 0.83)	0.98 (0.83, 1.14)
	Conscientiousness	0.78 (0.73, 0.85)	0.92 (0.77, 1.1)	0.96 (0.83, 1.11)
	Extraversion	1.14 (1.06, 1.24)	1.22 (1.02, 1.47)	1.13 (0.95, 1.33)
	Agreeableness	1 (0.91, 1.08)	0.99 (0.83, 1.19)	1.2 (0.99, 1.46)
	Neuroticism	1.05 (0.96, 1.16)	1.1 (0.9, 1.34)	1.21 (1.01, 1.45)
	External Locus of Control	1.1 (1, 1.21)	1.43 (1.17, 1.76)	1.06 (0.88, 1.28)

	Variable	Class 2	Class 3	Class 4
	Risk Behaviour	1.5 (1.37, 1.64)	2.9 (2.29, 3.68)	1.21 (1.02, 1.44)
	Empathy	0.77 (0.69, 0.84)	0.61 (0.5, 0.74)	0.88 (0.73, 1.07)
	Self-Isolating	0.65 (0.46, 0.93)	0.52 (0.21, 1.28)	1.12 (0.68, 1.84)
	Psychiatric Disorder	0.85 (0.67, 1.06)	1.17 (0.75, 1.85)	0.95 (0.57, 1.56)
Long-Term Conditions (ref. 0)	1	0.74 (0.62, 0.88)	0.72 (0.48, 1.08)	0.93 (0.69, 1.25)
	2+	0.6 (0.48, 0.76)	0.95 (0.61, 1.5)	0.89 (0.59, 1.34)

Table S4: Results of ordinal regression models predicting compliance behaviour (column) by individual characteristics. Models use multiply imputed data (40 datasets).

	Variable	Social Distance	Indoor Mixing	Outdoor Mixing	Wear Mask	Sanitise Hands	Guidelines (Other)
	Confidence in Government	1.44 (1.36, 1.54)	1.07 (0.99, 1.15)	1.05 (0.98, 1.13)	1.77 (1.57, 2.01)	1.11 (1.04, 1.18)	1.55 (1.45, 1.65)
COVID-19 Tier (ref. Lockdown)	Tier 3	1.17 (1.05, 1.3)	1.09 (0.96, 1.24)	1.26 (1.11, 1.42)	0.66 (0.53, 0.82)	1.05 (0.94, 1.16)	1.14 (1.02, 1.28)
	Tier 2	0.95 (0.86, 1.06)	0.97 (0.86, 1.1)	1.32 (1.17, 1.5)	0.81 (0.66, 1.01)	0.92 (0.83, 1.02)	1 (0.89, 1.11)
	Tier 1	0.95 (0.75, 1.21)	0.84 (0.64, 1.08)	1.19 (0.9, 1.58)	0.59 (0.38, 0.93)	1.14 (0.91, 1.43)	1.02 (0.79, 1.32)
	Wales	1 (0.86, 1.17)	0.82 (0.69, 0.98)	0.98 (0.83, 1.16)	0.77 (0.57, 1.04)	1.01 (0.87, 1.17)	0.92 (0.79, 1.09)
	Northern Ireland	0.93 (0.75, 1.17)	0.78 (0.61, 0.99)	0.55 (0.44, 0.68)	0.61 (0.39, 0.95)	1.61 (1.29, 2.01)	0.7 (0.56, 0.88)
Country (ref. England/Wales/NI)	Scotland	1.11 (1, 1.23)	1.06 (0.93, 1.21)	0.92 (0.81, 1.04)	1.45 (1.17, 1.81)	1.02 (0.92, 1.13)	1.06 (0.94, 1.18)

	Variable	Social Distance	Indoor Mixing	Outdoor Mixing	Wear Mask	Sanitise Hands	Guidelines (Other)
Gender (ref. Male)	Female	0.92 (0.86, 0.98)	1.01 (0.93, 1.09)	1.21 (1.13, 1.31)	1.42 (1.25, 1.61)	1.25 (1.17, 1.33)	1.03 (0.96, 1.1)
Ethnicity (ref. White)	Non-White	1.07 (0.97, 1.18)	0.83 (0.74, 0.92)	0.79 (0.71, 0.89)	0.93 (0.78, 1.12)	1.37 (1.24, 1.51)	1.03 (0.93, 1.15)
Age (ref. 18-29)	30-45	2.01 (1.83, 2.22)	1.35 (1.21, 1.5)	1.03 (0.92, 1.15)	1 (0.82, 1.21)	1.52 (1.38, 1.67)	1.61 (1.46, 1.78)
	46-59	3.05 (2.75, 3.39)	1.51 (1.34, 1.69)	1.1 (0.97, 1.24)	1.08 (0.88, 1.32)	1.73 (1.56, 1.92)	2.15 (1.93, 2.4)
	60+	3.82 (3.41, 4.27)	1.56 (1.37, 1.77)	0.87 (0.76, 1)	1.36 (1.09, 1.7)	2.11 (1.89, 2.36)	2.23 (1.99, 2.5)
	Keyworker	0.89 (0.83, 0.95)	0.88 (0.81, 0.96)	0.82 (0.76, 0.89)	0.63 (0.56, 0.73)	1.2 (1.11, 1.29)	0.9 (0.84, 0.97)
Education (ref. GCSE or below)	A-levels or equivalent	1.03 (0.96, 1.11)	1.28 (1.17, 1.4)	1.4 (1.28, 1.52)	1.25 (1.09, 1.44)	0.9 (0.84, 0.97)	1.22 (1.12, 1.32)
	Degree or above	0.98 (0.9, 1.06)	1.31 (1.19, 1.44)	1.46 (1.33, 1.6)	1.25 (1.06, 1.47)	0.89 (0.82, 0.97)	1.19 (1.09, 1.31)

	Variable	Social Distance	Indoor Mixing	Outdoor Mixing	Wear Mask	Sanitise Hands	Guidelines (Other)
Income (ref. < £16k)	£16k - £30k	1.02 (0.92, 1.13)	1.07 (0.94, 1.22)	1.15 (1.02, 1.3)	1.43 (1.18, 1.72)	0.93 (0.83, 1.03)	0.97 (0.87, 1.08)
	£30k - £60k	0.91 (0.81, 1.01)	1.18 (1.03, 1.34)	1.25 (1.1, 1.43)	1.89 (1.55, 2.32)	0.97 (0.87, 1.08)	0.99 (0.87, 1.11)
	£60k - £90k	0.8 (0.69, 0.91)	1.1 (0.93, 1.31)	1.18 (1, 1.38)	2.11 (1.58, 2.81)	0.81 (0.7, 0.93)	1 (0.87, 1.16)
	£90k+	0.88 (0.75, 1.03)	0.98 (0.81, 1.18)	0.95 (0.79, 1.15)	1.71 (1.26, 2.32)	0.75 (0.64, 0.88)	0.97 (0.8, 1.17)
Living Arrangement (ref. Not alone, no child)	Not alone, with child	0.93 (0.86, 1.01)	0.92 (0.84, 1)	0.94 (0.86, 1.03)	0.92 (0.79, 1.07)	0.83 (0.77, 0.9)	0.87 (0.81, 0.95)
	Alone	0.83 (0.77, 0.9)	1.02 (0.93, 1.12)	1.04 (0.95, 1.14)	0.88 (0.76, 1.02)	0.75 (0.69, 0.81)	0.93 (0.86, 1.02)
Household Overcrowding (ref. <1 persons per room)	1+ person per room	1.1 (1, 1.21)	0.84 (0.76, 0.93)	0.83 (0.75, 0.92)	0.88 (0.74, 1.04)	1.2 (1.09, 1.31)	0.9 (0.82, 0.99)
	Nieghbourhood Crowding	0.98 (0.9, 1.07)	0.99 (0.89, 1.1)	1.03 (0.93, 1.14)	0.96 (0.79, 1.15)	0.91 (0.84, 1)	0.98 (0.9, 1.07)

	Variable	Social Distance	Indoor Mixing	Outdoor Mixing	Wear Mask	Sanitise Hands	Guidelines (Other)
Lockdown 1.0 Mental Health (ref. Same)	Worse	0.97 (0.88, 1.06)	0.96 (0.85, 1.07)	1.01 (0.9, 1.13)	0.8 (0.67, 0.96)	1.03 (0.94, 1.14)	1.01 (0.91, 1.11)
	Better	0.85 (0.72, 0.99)	0.87 (0.71, 1.06)	0.9 (0.73, 1.11)	0.68 (0.53, 0.89)	0.99 (0.82, 1.19)	1 (0.85, 1.19)
	Openness	1.35 (1.26, 1.44)	1.2 (1.12, 1.3)	1.14 (1.06, 1.23)	1.15 (1.02, 1.3)	1.28 (1.21, 1.37)	1.23 (1.15, 1.32)
	Conscientiousness	1.36 (1.27, 1.45)	1.15 (1.07, 1.24)	1.16 (1.08, 1.25)	0.98 (0.86, 1.1)	1.45 (1.36, 1.54)	1.28 (1.2, 1.37)
	Extraversion	0.91 (0.85, 0.97)	0.75 (0.69, 0.81)	0.65 (0.61, 0.7)	0.85 (0.75, 0.96)	1.1 (1.03, 1.17)	0.92 (0.86, 0.98)
	Agreeableness	1.03 (0.96, 1.1)	0.92 (0.85, 1)	0.89 (0.82, 0.96)	1.26 (1.1, 1.43)	1.06 (0.99, 1.14)	1.1 (1.03, 1.19)
	Neuroticism	0.92 (0.85, 1)	0.85 (0.77, 0.93)	0.83 (0.76, 0.91)	1.19 (1.02, 1.39)	1.2 (1.11, 1.3)	0.96 (0.88, 1.05)
	External Locus of Control	0.8 (0.73, 0.87)	0.88 (0.78, 0.98)	0.82 (0.74, 0.91)	0.6 (0.5, 0.72)	0.78 (0.71, 0.86)	0.78 (0.7, 0.87)

	Variable	Social Distance	Indoor Mixing	Outdoor Mixing	Wear Mask	Sanitise Hands	Guidelines (Other)
	Risk Behaviour	0.49 (0.45, 0.54)	0.58 (0.52, 0.64)	0.58 (0.52, 0.64)	0.46 (0.37, 0.56)	0.59 (0.54, 0.65)	0.44 (0.4, 0.49)
	Empathy	1.48 (1.34, 1.62)	1.13 (1.01, 1.26)	1.24 (1.1, 1.39)	1.65 (1.39, 1.95)	1.58 (1.44, 1.75)	1.51 (1.36, 1.67)
	Self-Isolating	1.62 (1.4, 1.87)	1.36 (1.12, 1.64)	1.3 (1.09, 1.56)	0.81 (0.63, 1.05)	1.19 (1.03, 1.38)	1.28 (1.1, 1.5)
	Psychiatric Disorder	1.06 (0.98, 1.16)	1.11 (1.01, 1.23)	1.13 (1.02, 1.25)	0.87 (0.74, 1.02)	0.93 (0.85, 1.01)	1.09 (0.99, 1.19)
Long-Term Conditions (ref. 0)	1	1.34 (1.25, 1.44)	1.21 (1.11, 1.31)	1.12 (1.04, 1.21)	0.9 (0.78, 1.03)	1.2 (1.12, 1.29)	1.21 (1.12, 1.3)
	2+	1.48 (1.35, 1.63)	1.29 (1.15, 1.45)	1.11 (1, 1.24)	0.79 (0.67, 0.94)	1.43 (1.3, 1.57)	1.37 (1.23, 1.51)

References

1 Hale T, Angrist N, Cameron-Blake E, *et al.* Oxford COVID-19 Government Response Tracker. Oxford: : Blavatnik School of Government 2020. https://www.bsg.ox.ac.uk/research/research-projects/coronavirus-government-response-tracker (accessed 22 Oct 2020).

2 Soto CJ, John OP. The next Big Five Inventory (BFI-2): Developing and assessing a hierarchical model with 15 facets to enhance bandwidth, fidelity, and predictive power. *J Pers Soc Psychol* 2017;**113**:117–43. doi:10.1037/pspp0000096

3 Hirschman C, Almgren G. University of Washington - Beyond High School (UW-BHS): Version 5. 2012. doi:10.3886/ICPSR33321.V5

4 Dohmen T, Falk A, Huffman D, *et al.* INDIVIDUAL RISK ATTITUDES: MEASUREMENT, DETERMINANTS, AND BEHAVIORAL CONSEQUENCES. *J Eur Econ Assoc* 2011;**9**:522–50. doi:10.1111/j.1542-4774.2011.01015.x

5 Davis MH. Measuring individual differences in empathy: Evidence for a multidimensional approach. *J Pers Soc Psychol* 1983;**44**:113–26. doi:10.1037/0022-3514.44.1.113
